# The Ilizarov external fixator - a useful alternative for the treatment of proximal tibial fractures A prospective observational study of 30 consecutive patients

**DOI:** 10.1186/1471-2474-14-11

**Published:** 2013-01-07

**Authors:** Telmo Ramos, Carl Ekholm, Bengt I Eriksson, Jón Karlsson, Lars Nistor

**Affiliations:** 1 Department of Orthopaedics, Central Hospital (Kärnsjukhuset), Skövde SE-541 85, Sweden; 2 Department of Orthopaedics, Sahlgrenska University Hospital, Sahlgrenska Academy at Gothenburg University, Mölndal SE-431 80, Sweden

**Keywords:** Proximal tibial fractures, Ilizarov method, External fixation

## Abstract

**Background:**

In dislocated proximal tibial fractures, the most frequently used treatment is ORIF with screws and plates. Minimally-invasive techniques using external fixation are an alternative. The aim of this study was to analyse the clinical and radiological results using the Ilizarov technique in both uni- and bicondylar tibial fractures.

**Methods:**

Thirty consecutive patients with isolated fractures of the proximal tibia were treated with the Ilizarov technique, 11 Schatzker I-IV with 2–3 rings and 19 Schatzker V-VI with 3–4 tibial rings and a femoral, hinged, two-ring extension. Unrestricted weight-bearing was allowed. Pre and post-operatively, conventional radiographs, computerized tomography scans, post-operative pain assessments and complications were evaluated. The knee function was evaluated with the EQ-5D, NHP and KOOS scores, as well as self-appraisal.

**Results:**

All the fractures healed. Twenty-five patients achieved a range of motion better than 10-100º. The type I-IV fractures had a shorter operating time and hospital stay, as well as better knee flexion, and the self-appraisal indicated that they tolerated the treatment better. Pin infections occurred in 4% of the pin sites, but only two patients required debridement. Two patients developed compartment syndrome and underwent fasciotomy. No patient complained of functional knee instability. Two patients underwent a total knee arthroplasty because of residual pain. The overall result was judged as satisfactory in twenty-seven patients.

**Conclusions:**

The Ilizarov method produces a good clinical outcome and is a valuable treatment alternative in proximal tibial fractures of all types.

## Background

The goal of the treatment of tibial plateau fractures is to achieve a stable, well-aligned, mobile, pain-free joint and to minimise the risk of post-traumatic osteoarthritis [1,2].

In non-osteoporotic proximal metaphyseal tibial fractures of the Schatzker I-IV and AO/OTA types B and C1, open reduction and internal fixation (ORIF) using screws and plates is the recommended treatment. Fractures of the Schatzker V -VI and AO C2 and C3 types have also previously been treated in the same manner, but more recently the Ilizarov circular fixator is also considered to be an established treatment alternative [3,4].

Fractures of the articular surface of the tibia, even in fractures with minimal joint extension, are usually the result of a high-energy direct blow [5]. Because of the type of trauma involved and the relatively high frequency of major soft-tissue injuries [6] the complication rate is high, regardless of treatment [7]. The relatively large surgical incisions that are used for internal fixation also add a considerable risk of soft-tissue complications [8].

If the classic Ilizarov technique is used according to the original recommendations [9,10], the reduction and fixation of the fracture fragments can be made with almost no soft-tissue exposure and blood loss. This technique does not leave screws and plates when the fracture has healed. The fixator also allows for the adjustment of the alignment and for compression/distraction both during and after surgery. Another advantage when it comes to using the Ilizarov technique is that the fixation is stable enough to allow early weight-bearing. [11,12], which is the rationale for using the Ilizarov method in unicondylar fractures. In the communited bicondylar high energy fractures the rationale is the same and, in addition, there is no need to use a staged protocol.

Plate fixation and circular external fixation similar to the Ilizarov technique, were compared in a randomised, multicentre study of 83 displaced Schatzker V-VI fractures [13]. Both techniques produced satisfactory fracture reduction, but the number and severity of complications was greater with ORIF. In a review, Mahadeva et al. [14] compared internal and hybrid external fixation in Schatzker type VI tibial plateau fractures. The number of complications were larger in the group treated with ORIF, but, due to the limited number of reports (five), the differences were not statistically significant. To date, the rationale for treating unicondylar fractures is osteosynthesis with screws or plates, percutaneously if possible. The only report we have found in the English literature on Ilizarov applications in unicondylar fractures is by Watson et al. [15]. They included fourteen high-energy fractures treated with a combination of screws/clamps and the Ilizarov techniques (three-ring fixator, half-pins) and reported excellent results.

At our hospital, which is an educational institution, the Ilizarov external fixator was gradually introduced by one experienced trauma surgeon (TR) for displaced proximal tibial fractures in 2002 and, since 2005, it has been the preferred treatment for both unicondylar (Schatzker I-IV) and bicondylar fractures (Schatzker V-VI). The aim of the present study was to compare prospectively the clinical outcome and radiological healing and how the patients experienced the treatment in the two subgroups (unicondylar vs. bicondylar).

## Methods

The selection criteria in this study were as follows: Patients aged 18–75 years, with tibial plateau fractures displaced more than 5 mm and/or instability when the knee was stressed in varus or valgus, admitted to the Emergency Department at Skaraborg Central Hospital (Kärnsjukhuset) in Skövde; a referral trauma centre for a population of approximately 280.000 inhabitants. Only patients with isolated fractures and without disorders affecting gait, who were able to understand and follow instructions in Swedish, were enrolled after written informed consent for participation in the study. Between January 2005 and December 2009, 40 patients fulfilled the inclusion criteria. One individual refused to participate and nine patients were treated outside the protocol when the study supervisor was on leave. The remaining 30 patients were included in this prospective follow-up study. Their median age was 51 years (range 18–74), 12 were women and 18 men. Four patients were smokers. The cause of the injury was motor-vehicle accidents in 10 patients, falls in 13, horse riding accidents in 4, work accidents in 2 and a blow (assault) in 1 patient. Two patients were referred from another hospital with a provisional external fixation.

The pre-operative radiographs were supplemented with computerized tomography scans in 26 patients. Eight fractures also had a diaphyseal extension. There were no open fractures. The fractures were classified according to Schatzker [16]. Eleven patients (7 women and 4 men) had type I-IV fractures and 19 (11 women and 8 men) V-VI type. There were no significant demographic differences between the groups. The cause of injury related to the type of fracture is shown in Table 1. The patients were scheduled for early surgery. Sixteen patients were operated on within two days of the accident and the rest between 3 to 11 days. The median time between injury and surgery was 2 days (range 0–11).

**Table 1:** Details of all patients treated with the Ilizarov application and fractures types

**Case**	**Age**	**Mechanism of injury**	**Schatzker**	**AO**	**Energy type**
1	54	Fall	V	C3	High
2	38	Traffic	VI	C3	High
3	74	Fall	VI	C3	Low
4	18	Riding	II	B3	High
5	44	Traffic	III	B2	High
6	50	Work	I	B1	High
7	57	Fall	II	B3	Low
8	54	Fall	VI	C3	Low
9	60	Fall	VI	C3	High
10	34	Assault	II	B3	Low
11	59	Fall	VI	B3	Low
12	60	Fall	VI	C3	Low
13	35	Traffic	V	C3	High
14	58	Fall	II	B3	Low
15	62	Fall	II	B3	Low
16	70	Traffic	II	B3	High
17	43	Traffic	VI	C3	High
18	64	Fall	VI	C3	Low
19	63	Riding	II	B3	Low
20	53	Traffic	VI	C3	High
21	34	Traffic	VI	C3	High
22	27	Work	VI	C2	High
23	20	Traffic	VI	C1	High
24	62	Fall	VI	C3	Low
25	44	Riding	VI	C1	Low
26	44	Fall	IV	B1	Low
27	24	Riding	II	B3	Low
28	65	Fall	VI	C3	Low
29	43	Traffic	VI	C3	High
30	50	Traffic	VI	C3	Low

The surgery was performed without a tourniquet on a traction table with the foot fixed in a shoe. An arthrocentesis was made to reduce the intra-articular pression. Biplane fluoroscopy was used during reduction, pin insertion and assembly of the frame. The axial reduction was achieved with traction. The joint surface was reconstructed if necessary, using closed pressure with percutaneously inserted elevators, reduction forceps or/and wires with olives. Arthrotomy or arthroscopy was not used. Subchondral defects were packed with calcium sulphate bone pellets (Osteoset® or β-tri calcium phosphate ChronOs®) in 18 patients. The proximal ring was placed at the level of the fibular head. Additional stability was achieved using 1.8 mm wires parallel to the articular surface with posts secured on the rings (drop-wire techniques). The wires were tensioned to at least 110 Kg. Depending on the complexity of the fracture, another one to three rings were fixed with two to three wires in the tibia, and they were then connected with steel rods (Smith & Nephew, Memphis, Tennessee, USA). In Schatzker type V-VI fractures, two rings in the distal femur were added to the construction in 16 patients with hinged rods over the knee. No additional osteosynthesis was used. Nineteen operations were performed by TR who also supervised residents in the remaining 11. No post-operative corrections were needed.

Cloxacillin (2 g) was used as infection prophylaxis starting pre-operatively. Low-molecular heparin prophylaxis was given from the day of admission until 10 days after leaving the hospital. During the first 24 hours after surgery, all patients had a post-operative continuous i.v. analgesia (PCA) pump with morphine/ketobemidon.

The “Kurgan protocol” [17] was used for postoperative pin site dressings and the Checketts-Otterburns classification [18] was used to describe pin infections.

Physiotherapy was started immediately after the operation to maintain knee and ankle motion and the patients were allowed to start unrestricted weight-bearing.

The femoral extension was used in 16 of the 19 type V-VI fractures and was removed at six weeks. The fractures were regarded as being healed when antero-posterior and lateral radiographs showed a bridging callus of three of four cortices and/or the fracture was stable when stressed manually and the patients were able to walk without pain after the connecting rods had been removed. The fixators could then be removed without anaesthesia, for type I-IV fractures after 11 weeks (range 6–16) and for type V-VI at 12 weeks (range 8–20) post-operatively.

All the patients were followed up clinically after two, four, eight and 12 weeks and finally at one year. Radiography was performed at the same intervals. Additional clinical and radiographic assessments were made when necessary to evaluate fracture healing.

Pain and patient satisfaction were registered using a visual analogue scale (VAS) at four and 12 weeks and at the one year follow-up. The Swedish versions of the EuroQol (EQ-5D) [19] and the Nottingham Health Profile (NHP) [20,21] were used for patient self-appraisal at the same time intervals. The clinical one-year post-operative outcome, including the ROM and manual testing of stability in varus and valgus, was assessed by an independent physiotherapist. The KOOS questionnaire [22,23] was added to the follow-up between one and five years post-operatively and in those patients in whom the observation period exceeded one year. Pain (VAS), EQ-5D and NHP questionnaires were repeated.

The post-operative radiographs were evaluated by one of the authors (TR) and separately by an independent surgeon according to the criteria formulated by Rasmussen [24].

1. The articular step-off – the maximal depression or displacement of the articular surface in an axial direction on antero-posterior and lateral projections.

2. The condylar widening – measured in comparison with the ipsislateral femoral condyles.

3. The plateau tilt – the angle in the varus or valgus direction as measured on the antero-posterior projections perpendicular to the long axis of the tibia.

### Statistical analysis

The median values and 95% confidence interval (CI) or range are given. Further statistical comparisons between the groups are not meaningful as the number of patients is small and they would only reflect differences that could be anticipated.

The study was approved by the regional ethical review board at Sahlgrenska University Hospital in Gothenburg (ID. 400–04).

## Results

The comparison of the two subgroups, Schatzker I-IV and Schatzker V-VI, is shown in Table 2.

**Table 2:** Treatment timing in the two subgroups

	**Schatzker I-IV (n = 11)**		**Schatzker V-VI (n = 19)**	
	**Median**	**CI**	**Median**	**CI**
Surgery delay (days)	3	(1–11)	2	(1–9)
Operation time (min)	130	(100–165)	223	(164–240)
Hospital stay (days)	4	(3–6)	9	(7–11)
External fixation (weeks)	11	(7–16)	12	(10–15)

The median surgical time, including the time for assembling the frame peri-operatively, was lower for the Schatzker I-IV (130 minutes, range 92–117) than for the Schatzker V-VI fractures (223 minutes, range 97–275).

The total amount of morphine/ketobemidon (PCA pump) varied between 7 and 77 mg (median 46 mg). The demand for additional analgesics was low.

All the patients were allowed full, unrestricted weight-bearing from the first post-operative day and were discharged directly to their homes when they managed to walk using crutches and independently climb stairs. The Schatzker I-IV group had a shorter hospital stay, 4 days (range 3–9), than the Schatzker V-VI; 9 days (range 3–13).

The observed complications are shown in Table 3. Two patients with Schatzker VI fractures developed compartment syndromes (case 2 Schatzker VI/AO C3 and case 28 VI/C2). In the first patient, the compartment syndrome was masked by an over consumption of opiates and the patient did not undergo fasciotomy until one day after the initial operation. He had a muscle necrosis of the lateral compartment of the leg and permanent peroneal nerve palsy. The other patient underwent fasciotomy immediately after the application of the fixator. He developed a fistula in the fasciotomy wound, which required excision, but healed without sequelae.

**Table 3:** Complications in all fractures

**Complications**	**n = 30**
Compartment syndrome	2
Deep vein thrombosis	1
Secondary dislocation	2
Pin-site infections	16
Pin-track infection	2
Osteomyelitis	0
Nerve injury	0

A total of 113 rings and 321 wires were used, constituting 642 potential pin-infection sites. Sixteen patients had 25 minor pin site infections, Checketts-Otterburns grades 1–3, all of which subsided with short-term oral antibiotics and two had pin tract infections grade 4 that healed after soft-tissue debridement. There were no clinical or radiological signs of osteomyelitis or septic arthritis in any patient.

One patient developed a distal DVT with the fixator still in place two months after the injury.

At the one-year follow-up, 27 patients had an extension deficit of less than 10°. The patients with Schatzker I-IV fractures had better knee flexion (140°, range 86–156) than those with Schatzker V-VI fractures (120°, range 83–148), see Table 4. Three patients were able to flex their knee less than 90° and they also had extension deficits of more than 10°. Four of the five patients with reduced knee flexion had Schatzker type V or VI fractures. Ankle motion was not affected. Two knees were mobilized under epidural anesthesia postoperatively at five months (case 30) and seven months (case 29).

**Table 4:** Range of motion at one year, median (range) in the two subgroups

	**Schatzker I-IV (n = 11)**				**Schatzker V-VI (n = 19)**			
	**Uninjured**		**Injured**		**Uninjured**		**Injured**	
Knee flexion	146°	(124-160°)	140°	(86-156°)	140°	(120-154°)	120°	(83-148°)
Knee extension	0°	(0-8°)	3°	(0-17°)	0°	(0-8°)	0°	(0-20°)

Residual knee laxity was observed in three patients (cases 19, 20 and 28), but no patient complained of functional instability of the knee. The radiological results at the one-year follow-up were good in 27 patients according to the criteria formulated by Rasmussen [24]. The bone substitutes were all at least partially integrated and there were no signs of adverse reactions. Detailed results for the patients who had instability or significant radiological deformity (residual deformity of 10 mm articular depression and/or condylar widening of more than 10 mm and/or valgus and varus more than 10°) are summarised in Table 5 and compared with the KOOS values and patient satisfaction. Two patients with increasingly severe pain (case 1 and case 24) underwent total knee arthroplasty 1.5 and two years after the initial fracture treatment.

**Table 5:** Outcome at one year in patients with instability and/or significant residual radiological deformity

**Case**	**Stable/Unstable**	**Tilt degrees**	**Articular depression mm**	**KOOS pain**	**KOOS symptom**	**KOOS ADL**	**KOOS Sport**	**KOOS QoL**	**VAS Satis-faction mm**	**VAS Pain mm**
8	S	13 varus	10	38.8	64.2	42.6	0	25	69	23
16	S	8 valgus	10	91.6	71.4	82.3	35	68.8	57	9
19	US	3 valgus	7	-	-	-	-	-	13	20
20	US	1 varus	2	86.1	64.2	88.2	15	62.5	6	8
26	S	12 varus	0	88.9	92.8	97.0	80	62.5	14	13
28	US	5 valgus	0	91.6	46.4	61.7	25	50	27	-

The pain (VAS), patient satisfaction (VAS), EQ5D and the NHP total score outcomes at different time intervals are shown in Table 6. The differences between the Pain (VAS), EQ-5D, NHP values at one year and the KOOS questionnaire were not significant. The EQ-5D values and NHP total scores show that the overall function was severely affected at four weeks. However, there were no differences between the subgroups. The knee function improved more rapidly in patients with Schatzker I-IV fractures in those with the Schatzker V-VI. Good knee function was registered first at the one-year follow-up when there were no differences between the groups.

**Table 6:** Outcome according to patients’ self-appraisal controls in the two subgroups

**Median with 95% CI**	**Time of assessment**	**Schatzker I-IV**		**Schatzker V-VI**	
**Pain (VAS)**	4 weeks	20	(0–50)	28	(24–47)
	12 weeks	7	(0–45)	25	(9–29)
	1 year	9	(0–21)	16	(1–23)
**Patient satisfaction (VAS)**	4 weeks	9	(3–28)	28	(7–42)
	12 weeks	8	(5–48)	14	(6–30)
	1 year	13	(0–22)	13	(6–23)
**NHP total**	4 weeks	5.3	(3.5-36.8)	37.3	(15.7-46.8)
	12 weeks	1.8	(0–17.4)	20.2	(11.2-34.5)
	1 year	1.8	(0–11.4)	7.4	(1.8-19.3)
**EQ5D**	4 weeks	0.66	(0.29-0.81)	0.59	(0.29-0.62)
	12 weeks	0.76	(0.62-1.0)	0.62	(0.29-0.69)
	1 year	0.89	(0.69-1.0)	0.80	(0.69-0.85)

**Figure 1:**
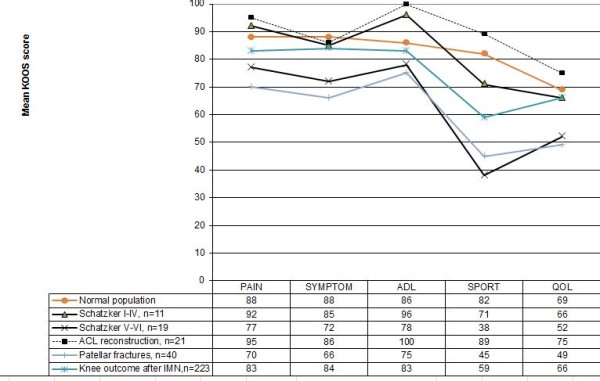
**The KOOS subscores compared with the literature**[22,75,76].

The KOOS values are shown in Figure 1 and compared with the results from the literature.

Comparison with other studies regarding complications and outcome is given in Tables 7 and 8.

**Table 7:** The trial with the cohort subgroup with Schatzker I-IV type of fractures compared with the literature

	**Present study**	**Sament et al. 2012**	**Allam et al. 2011**	**Watson et al. 1998**	**Keogh et al. 1992**	**Koval et al. 1992**
**Study design**	P	P	P	P	P	P
**Number of patients**	11	36+(20 S-V)	29	14 (S I-II)	13 (1SV)	20 (S-V)
**High-energy**	4	50	NM	14	NM	20
**Time to definitive surgery (days)**	2	2	NM	12	NM	4
**Intervention**	CEF	IF+cast	IF+cast or brace	ORIF+CEF	IF+cast or brace	IF
**Full weight-bearing (weeks)**	immediate	12	8-12	12	8-12	12
**Follow-up (months)**	12	34	30	19	17	16
**Knee ROM injured side**	0-140	90% > 90	NM	0-108	NM	0-128
**Healing (weeks)**	11	12	9	29	NM	12
**Compartment Syndrome**	0	0	0	0	0	0
**Deep Vein Thrombosis**	0	0	0	0	1	0
**Deep Infection**	0	0	0	0	0	0
**Reoperations**	Arthroscopy:1	0	0	Staged protocol in 8 patients	Implant removal:7	NM
	TKR:0					
				Skin graft:1		
				Delayed wound closure:1		
				Average 3 procedures (exclusive the frame removal)		
**Functional outcome**	VAS Pain	Rasmussen score		Knee Society score	Rasmussen score	Motion
	VAS Satisfaction					Pain
						Deformity
	NHP					Ambulation
	EQ5-D					Return to work
	KOOS					

P = Prospective, R = Retrospective, NM = Not mentioned, CEF = Circular External Fixator, IF = Internal Fixation.

Unilat = Unilateral Extern Fixation, TKR = Total Knee Replacement, S = Schatzker fracture type, ROM = Range Of Motion.

**Table 8:** The trial with the cohort subgroup with Schatzker V-VI type of fractures compared with the literature

	**Present study**	**Yu et al. 2009**	**Catagni et al. 2007**	**Lee et al. 2007**	**Phisitkul et al. 2007**	**Boldin et al. 2006**	**Canadian Orthopaedic Trauma Society 2006**	**Oh et al. 2006**	**Egol et al. 2005**	**El-Barbary et al. 2005**	**Cole et al. 2004**	**Ricci et al. 2004**	**Dendrinos et al. 1996**	**Zecher et al. 1996**	**Yang et al. 1995**	**Marsh et al. 1995**
**Study design**	P	P	R	R	R	P	Rand	P	P	R	R	P	P	P	P/R	P
**Number of patients**	19	54	59	35	37	25	82	23	53	29	87	28	24	21	22/22	20
**High-Energy**	14	54	59	35	37	18	NM	NM	53	29	75	NM	24	21	40	20
**Time to definitive surgery (days)**	2	10	NM	12	13	6	4	11	9	NM	7	NM	2	NM	NM	2
**Intervention**	CEF	ORIF	CEF + IF	ORIF	ORIF	ORIF	40 ORIF/42 CEF	ORIF	ORIF	CEF + IF	ORIF	ORIF	CEF + IF	CEF + IF	ORIF + cast/CEF + IF	Unilat + IF
**Full weight-bearing (weeks)**	immediate	19	immediate in 40 patients with femoral extension	12	16 patients: 11	NM	NM	14	12	6	13	Extra-articular fractures: 6–8	14	NM	12/08/12	4-8
					19 patients: 15											
												Intra-articular fractures: 8-12				
**Follow-up (months)**	12	24	21	3-26	49	36	24	25	16	27	14	23	37	14	21–208	38
**Knee ROM ° injured side**	0-120	0-108	0-119	0-135	0-112	0-117	0-120 No differences	0-123	0-106	0-112	0-120	NM	90% >110	>90		0-130
**Healing (weeks)**	12	15	29	16	12	11	NM	19	NM	NM	13	NM	14	0	16/20	15
**Compartment syndrome**	2	0	0	0	1	1	3	0	10	0	0	4	3	7	0	0
**Deep Vein Thrombosis**	1	0	2	0	0	0	0	0	0	0	0	0	8	1	0	0
**Deep infection**	0	2	0	2	8	0	18% ORIF	0	2	0	2	0	0	0	5/1	9
**Reoperations**	TKR: 2	TKR: 2	0	Irrigation + drainage: 2	Staged protocol in 28 fractures with cast, EF and later ORIF	TKR: 2	Removal of EF: 27		Staged protocol for all patients with knee spanning fixation and later ORIF	NM	Quadricepsplasty: 2	Stage protocol with EF before ORIF: 2	Fasciotomy: 3	Revision with ORIF: 2	Soft-tissue flap: 8/1	Arthrotomy + drainage: 2
	Knee manipulation: 2	Plate removal: 2		Soft tissue flap: 2	Irrigation + drainage: 8	Fasciotomy: 1	Total 37/16		Soft tissue flaps: 4		Loss of fixation: 2	TKR:1				Revision of fixator: 3
	Fasciotomy: 2 Arthroscopy: 1				Plate removal: 6	Bone graft: 1	TKR: 2/1		Plate removal: 4		Irrigation + drainage: 5	Fasciotomy: 2				
					Amputation: 1		Knee manipula- tion: 3/2		Incision + drainage: 1		Bone graft: 1	Plate removal: 1				
					Fasciotomy: 3		Incision + drainage: 8/2		Bone graft: 2		Amputation: 1					
					Skin graft: 1		Skin graft: 5/2		Quadricepsplasty: 1		Change of implant: 3					
							Screw removal: 0/6		Knee mani- pulation + arthroscopy: 2		Plate removal: 10					
							Plate removal: 8/0									
							Amputation: 1/0									
							Soft-tissue flap: 4/0									
							Other: 6/3									
**Functional outcome**	VAS Pain	HSS score	ASAMI score	0	0	HSS score	SF-36 WOMAC HSS knees	Rasmussen score	WOMAC	Knee Society score	0	Lower Extremity Measure	Honkonen score		0	SF-36
	VAS Satisfaction	Lysholm score				Knee Society score										Iowa
	NHP	Knee Society scoer														Knee score
	EQ5-D															
	KOOS															

P = Prospective, R = Retrospective, Rand = Randomiserade, NM = Not mentioned, CEF = Circular External Fixator, IF = Internal Fixation,

ORIF = Open Reduction and Internal Fixation, Unilat = Unilateral Extern Fixation, TKR = Total Knee Replacement, ROM = Range of Motion.

## Discussion

The most important finding in this study was that, in both unicondylar (Schatzker I-IV) and bicondylar fractures (Schatzker V-VI), the Ilizarov fixation allowed early weight-bearing without jeopardising the fracture stability and healing.

Maripuri et al. [25] claimed that the Schatzker classification was superior to the AO [26] and the Hohl and Moore [27] classification in terms of both inter-observer reliability and intra-observer reproducibility. However, they also concluded that none of the classifications was able fully to describe all fracture types. In the present study, the Schatzker classification was used to differentiate between two biomechanically different fracture subsets, one with continuity between a part of the articular surfaces and the diaphysis (I-IV types) and one without such continuity (V-VI types). Most unicondylar tibial fractures are caused by a forced varus or valgus load. In bicondylar tibial fractures, there is also an axial load resulting in a combination of depression of the articular surface, metaphyseal crush and shearing of one or both condyles. Vertical displacement is possible because there is no shaft below the fragment, which creates a shear vector. With the “olive wires” in the Ilizarov ring fixator; these forces are counteracted, holding the condyles together, which creates a relatively stable joint surface configuration that can be fixed to the tibia distally of the fracture. The distinction between uni- and bicondylar fracture is important, because, in fracture types I-IV, there is a risk of dislocation of the fractured part of the articular surface relative to the diaphysis when loaded. Because of the discontinuity between the articular fragments and the diaphysis in the V-VI fractures, compressive forces will not normally increase the risk of displacement of the articular surfaces.

As expected, the operating time was longer for the more complex fractures. In spite of this, the operating time in the present study compares favourably with that of Lee et al. [28] who operated on thirty-six tibial plateau fractures using the less invasive stabilisation system (LISS); their mean operation time was 150 minutes. Pre-assembling the frame could reduce the time in the operating room but one important advantage of the Ilizarov technique is that it is an essentially closed method and if the surgical time is extended, the risk of wound contamination is low when compared with open plating of the tibial plateau [29].

The pain subsided rapidly and did not constitute a problem after the first 24 hours post-operatively. We have not found any report of a need for post-operative analgesia in these types of fractures, but the amount of analgesics in the PCA pump corresponds to that in patients with total knee arthroplasties in our hospital data base.

The reported incidence of joint capsule, ligament and meniscal injuries is high. Colletti et al. [6] analysed MRI findings in 29 tibial plateau fractures and found associated collateral ligament injuries in 55%, lateral meniscal tears in 45%, anterior cruciate ligament injuries in 41%, posterior cruciate ligament injuries in 28%, and medial meniscal tears in 21%. Gardner et al. [30] found that only 1% of tibial plateau fractures showed a complete absence of soft-tissue injuries, evaluated by MRI. These injuries can also be diagnosed arthroscopically [31]. However, even if recommended by some authors [32-35] there is no support for this in randomised trials [36]. The percutaneous treatment of fractures of the tibial plateau can be performed using arthroscopy or fluoroscopy to control the reduction of the joint surface. Lobenhoffer et al. [37] were not able to demonstrate any significant benefit from arthroscopy compared with fluoroscopic reduction in 168 patients with tibial plateau fractures. Ohdera et al. [38] found no significant difference between arthroscopic management of tibial plateau fractures compared with the open reduction method in terms of duration of operation, post-operative flexion, and clinical results in 28 patients. The arthroscopic procedure was only recommended in selected tibial plateau fractures. In the present series, it was possible to achieve an acceptable reduction according to the criteria formulated by Rasmussen [39] in most patients without the use of arthroscopy.

In a retrospective study, Park et al. [40] found a low rate (1.6%) of compartment syndromes requiring fasciotomy for proximal tibia fractures. However, in more complex fractures, the risk of compartment syndrome is considerably higher. For Schatzker type VI fractures, Stark et al. [41] found an overall risk of 27%, as well as a difference depending on whether or not the medial plateau was dislocated, 53 and 18% respectively. The incidence of compartment syndrome in the severe fractures (V and VI) in the present series was comparatively low; 2/19 patients; however the observed compartment syndromes were interpreted as a direct result of the fracture and the soft-tissue injury and not of the operation. In spite of the Ilizarov technique is beneficial with respect to soft-tissue injury, minimizing the risk of developing compartment syndrome; the frame should not prevent this salvage procedure when necessary.

Some studies support the staged protocol for proximal tibial fractures, especially if high energy fractures are present [42-45]. The Ilizarov method gives the advantage, independently of fracture pattern, to operate on all patients without delay. In this way, we were able to avoid disturbing the healing process with other further interventions to the soft-tissues which may delay rehabilitation.

Most treatment methods do not allow full weight-bearing in intra-articular proximal tibial fractures [46]. The mobilisation and degree of weight-bearing that is allowed is determined by the fracture displacement, method of treatment, and quality of aftercare [47,48]. In the present study, all the patients were allowed unrestricted weight-bearing without any signs of the reduction being compromised.

In earlier series, the infection rate after treating tibial plateau fractures with ORIF, varies from 6% to 87.5% [49-51]. The use of bilateral incisions and the reduction of the size of the implants have reduced this rate to 3 –8.4% [52-54]. Despite using a generally recommended staged protocol, Egol et al. [43] reported a deep wound infection rate of 5%. When comparing external devices in different locations, Parameswaran et al. [55] reported that ring fixators had the lowest incidence of infection. Using the Ilizarov technique, Catagni et al. [56] did not observe any deep infections in a series of 59 patients with Schatzker V-VI fractures. In the present series, the majority of observed infections were easy-to-treat superficial “pin-site” infections. Only two patients had “pin-tract” infections, and they could be treated without compromising the fixation or fracture healing.

The use of autogenous iliac crest bone grafts is associated with risk of increased morbidity from the donor site [57,58]. Good results have been reported in previous studies using bone graft substitutes in terms of the prevention of redislocation of the articular surface in tibial plateau fractures [59,60]. Beuerlein and McKee [61] found several studies reporting that calcium sulphate is an effective safe void-filler in bone defects after impacted fractures have been reduced. There is also evidence that the bioresorbable calcium phosphate is a better choice than autogeneous iliac bone grafts for the treatment of subarticular defects associated with unstable tibial plateau fractures [62,63]. At the one-year control, we were unable to detect any subsidence of the graft, which can be regarded as being at least partly integrated in all patients.

Conventional radiographs alone are not able to define union in internally fixed fractures with sufficient accuracy to enable their use as end-points of fracture healing. Generally, deciding when a fracture can be regarded as “healed” is difficult. In a recent study, Corrales et al. [64] reported a lack of consensus with regard to the definition of fracture healing. The surgeon’s ability to judge fracture union using chronological radiographs following internal fixation is estimated to be correct in approximately 70% [65]. The use of traditional external fixation methods, such as manual testing of fracture stability and/or pain response to weight-loading with the frame disassembled, can be added to the evaluation of the radiological healing. These tests could therefore be used to assess whether the fracture has healed sufficiently to allow the safe removal of the fixator and full, unprotected weight-bearing. Using these criteria, we had no refractures or increased deformities.

Several authors have discussed the degree of dislocation that can be accepted with remaining good knee function. The long-term results reported by Rasmussen [39] and Lansinger et al. [66] showed that a residual depression of up to 10 mm could be accepted if the knee was stable. In a 5-year follow-up on 109 fractures, Lucht and Pilgaard [67] reported that the functional outcome with a depression of <10 mm was acceptable. In terms of articular depression the recommended “acceptable” dislocation varies between 2 and 10 mm [68]. Marsh et al. [69] pointed out that the scientific basis for the different recommendations is generally weak. Giannoudis et al. [70] found that, in tibial plateau fractures, articular incongruities appear to be well tolerated. In addition to the articular depression, Rasmussen also found that instability and residual joint malalignment with varus and valgus angulations over 10° affected the outcome adversely. The residual displacements observed in the present series are within these limits in all but three patients. No one of the three patients with asymptomatic knee laxity had a valgus plateau tilt exceeding five degrees.

Knee stiffness is a common problem after tibial plateau fracture surgery [7] Gaston et al. [71] reported that, at one year, patients with tibial plateau fractures still ran a risk of 20% risk of knee stiffness, defined as flexion of less than 100° and an extension deficit of less than 5°. However, good results have been achieved with hybrid or ring fixators [72,73] and the results in the present study compare favorably with these. Even in the complex fractures requiring a hinged extension to the femur, only four of 15 patients had knee flexion of 90° or less.

It has been shown that individuals with proximal tibial fractures have substantial residual limb-specific and general health deficits even at two years of follow-up [13]. When we started the present study self-appraisal scores were rarely used in fracture patients. In recent years there has been an increasing interest in the patients opinion about the outcome. However there is no consensus of which score to use. As showed in Tables 7 and 8 different self-appraisal scores are being used or scores that are a mixture of the patients and the surgeons opinion. A previous evaluation of NHP scores in a prospective trial designed to study the effect of Ilizarov reconstruction of post-traumatic lower-limb deformities on general health status showed improvements equal to or better than the improvements reported for other orthopaedic procedures, including total joint arthroplasty [74]. The patients self-appraisals used in the present series (NHP, EQ5D, Pain-VAS, Satisfaction-VAS, KOOS) showed that the Ilizarov fixator was well tolerated and the overall restoration of function was good. Some residual pain was still present at the one-year control, which most probably reflects the severe nature of these fractures more than treatment failure.

Despite successful treatment and improvement in their outcomes, the KOOS subscores showed the lowest values for Sports and QOL activities, which is probably due to the fact that patients studied earlier with this score are commonly younger and more active than the patients enrolled in the present study. In two recently published studies KOOS have been used in the follow-up after intramedullary nailing of tibial shaft fractures and operated patellar fractures showing comparable results as in the present study [75,76]. Apart from this, patients with fractures type I-IV had results similar to patients after ACL reconstruction [22] and also the patients with type V-VI had acceptable results.

## Conclusions

The study confirms that the Ilizarov technique is a safe and effective method with a relatively low complication rate. It produces good results in both Schatzker type I-IV and Schatzker type V-VI fractures. The results are comparable to those previously reported with other techniques. Early and definite fixation is achieved with the Ilizarov technique, allowing immediate full weight-bearing, and the compliance is good. From a long-term perspective, the residual fracture displacements were within the range at which the risk of post-traumatic arthritis is low. The results from the present series indicate that the Ilizarov method is a valuable alternative in the treatment of both Schatzker I-IV and V-VI fracture types.

## Abbreviations

AO: Arbeitsgemeinschaft für Osteosynthesefragen; COTS: The Canadian Orthopaedic Trauma Society; ORIF: Open Reduction Internal fixation; ROM: Range of Movement

## Competing interests

The authors declare that they have no competing interests.

## Authors’ contributions

TR conducted the study and wrote the manuscript. JK and LN participated in the design of the study, which LN supervised. They both helped to analyse the results and revised the manuscript, together with CE and BE. All the authors agreed on the final content of the manuscript.

## Pre-publication history

The pre-publication history for this paper can be accessed here:

http://www.biomedcentral.com/1471-2474/14/11/prepub
